# Bacterial community analysis of floor dust and HEPA filters in air purifiers used in office rooms in ILAS, Beijing

**DOI:** 10.1038/s41598-020-63543-1

**Published:** 2020-04-14

**Authors:** Jianguo Guo, Yi Xiong, Taisheng Kang, Zhiguang Xiang, Chuan Qin

**Affiliations:** 1 Institute of Laboratory Animal Science, Chinese Academy of Medical Sciences & Peking Union Medical College, Beijing, 100021 China; 2 NHC Key Laboratory of Human Disease Comparative Medicine, Beijing, 100021 China; 3 Department of Food Science and Engineering, School of Chemistry and Chemical Engineering, Harbin Institute of Technology, Harbin, 150001 China

**Keywords:** Microbial ecology, Risk factors

## Abstract

Air purifiers with high-efficiency particulate air (HEPA) filters remove not only particulate matter but also airborne microorganisms in indoor environments. We investigated the bacterial community in HEPA filters (used for 1 year) and that in the floor dust of 12 office rooms in Beijing. We found that the viable bacteria proportion in the filter was significantly higher than that in the floor dust (*p* < 0.001). The Non-Metric Multi-Dimensional Scaling analysis showed that the bacterial communities in the filters and dust were significantly different (*p* = 0.001). The Chao1, Shannon–Wiener and phylogenetic diversity values in the filter were significantly higher than those in the dust (*p* < 0.001). The predominant bacterial classes in the filter were *Alphaproteobacteria* and *Actinobacteria*, whereas those in the dust were *Bacteroidia*, *Clostridia* and *Bacilli*. Human occupancy contributed more to the bacterial community in the filter than that in the dust. *Klebsiella* and *Alloprevotella* in the dust and filters positively correlated with the occupancy density. Soil bacteria contributed to a significantly higher proportion of the bacteria in the HEPA filter (*p* < 0.001). In contrast, human oral, indoor air and outdoor haze contributed to a higher proportion of the bacteria in the dust samples (*p* < 0.001, *p* < 0.01 and *p* < 0.05, respectively). As HEPA filters serve as an ecological niche for indoor bacteria, they should be carefully investigated during the assessment of indoor environmental health.

## Introduction

People spend the majority of their time indoors, wherein air pollution levels highly correlate with outdoor pollution levels^1–6^. In recent years, haze pollution, fine particulate matter (PM) in particular, has been reported to increase human mortality rates owing to the development of heart disease^7,8^, stroke, respiratory disease^9^ and lung cancer^10,11^. The level of air pollution in China is substantially higher than in many developed countries^12^. Moreover, its threat to public health has become a serious concern, particularly concerning haze events^13^. Thus, an effective technology is essential for the removal of PM from indoor environments during periods of haze. To this end, air purifiers with high-efficiency particulate air (HEPA) filters are widely used in the indoor environment, particularly in areas with heavy haze^14^.

People are exposed to various microbes in indoor environments. Many factors could affect indoor microbial composition, including human occupancy, occupant behavior, pets and outdoor environment^15–18^. The air purifier was mainly used to remove indoor PM, partly comprising microorganisms. Microbes in the air could be transmitted to the HEPA filter in the air purifier. Suzuki *et al*. (2003) collected swab samples from the air purifier filters in the houses of seven healthy children with varicella. They found the varicella-zoster virus was detected in 29% of filters in air purifiers by day 3^19^. Furthermore, Maus *et al*. (2001) studied the effect of the filters on the viability of microbes. They reported microbes could keep alive for a long time in the filter^20^. Majchrzycka *et al*. (2016) proved that the survivability of microorganisms on filter materials depends on the amount of accumulated moisture and microorganism type^21^. For example, *Staphylococcus aureus* survived well on filter materials with particular condition^21^. Under the working condition, microbes in the HEPA filter could be inspired into the air. Gore *et al*. (2003) reported that the old HEPA-filter vacuum cleaner markedly increases the inspired cat allergen in operation compared with baseline^22^. Spores were also detected downstream of the filters after a longer period of conditioning when ventilation was restarted^23^. These all indicated the microbes in the HEPA filter could be an important exposure source. Noris *et al*. reported the vast difference between HVAC filter and surface dust in an exploratory investigation in Austin^24^. However, the components of PM were distinctly different around the world. Surface PARTiculate mAtter Network collecting filter samples worldwide showed the chemical components of PM_2.5_ varied by more than an order of magnitude between sites^25^. Besides, one research involving 19 cities around the world showed up to 100-fold in both bacterial and fungal levels among cities^26^. So, the microbial communities in the air purifier are regional. Moreover, limited studies were carried out on the microbial community in HEPA filter in an air purifier, while most focused on microbes in HVAC filter^24,27–31^. Knowledge of microbes in HEPA filter in office was also lacked, except the concentration and the size distribution of microbes^32^. Settled dust (SD), which well reflects the microbial composition in indoor air^33–35^, was commonly used to explore the indoor microbial community^36^. It was reported that indoor dust could induce pulmonary inflammation^37,38^. Microorganisms in SD and HEPA filter in the purifiers both come from indoor air. Whether the microbial composition and source in the HEPA filter was similar to SD or should be considered as a new indoor ecological niche? Thus, developing a fundamental understanding of the origins and the characteristics of the microbial community in HEPA filter is a research priority in order to reduce the human health risk of exposure from air purifiers in indoor environments^39^.

Bacteria are the main microorganisms in indoor environments^40^, and indoor bacteria can be affected by differences in building design, ventilation, the environmental parameters^41–46^. Thus, in our study, we selected 12 office rooms with the same building design with natural ventilation. We investigated the characteristics of the bacterial communities, bacterial diversity and bacterial sources in the HEPA filters in air purifiers used in the indoor environment compared to floor dust collected in the corresponding rooms. Our results would help us to take appropriate measures to reduce the risk in the process of removing PM using air purifiers.

## Results

### Cell viability results of the bacteria in the filter and dust samples

**Figure 1:**
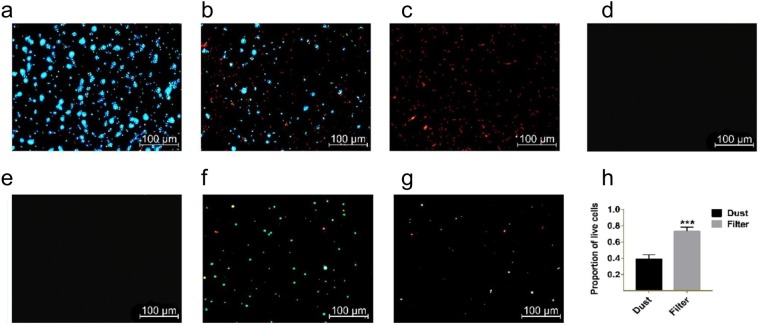
Proportion of the live cells in the dust and filter samples: 100% live cells of *E*. *coli* (**a**), 50% live cells of *E*. *coli* (**b**), 0% live cells of *E*. *coli* (**c**), filter sample without dyeing (**d**), dust sample without dyeing (**e**), filter sample after dyeing (**f**) dust sample after dyeing (**g**), and proportion of live cells in the dust and filter (**h**). Green, live cells; Red, dead cells. ‘*’: *p* < 0.05, ‘**’: *p* < 0.01, ‘***’: *p* < 0.001.

Live bacteria were stained blue, whereas dead bacteria were stained red. The feasibility of this method was verified using different concentrations of control *E*. *coli* samples (Fig. 1a–c). Samples without dye showed no fluorescent signal (Fig. 1d,e), indicating that no PM in the samples showed a fluorescent signal. The survival rates of the bacteria in the filter samples were significantly higher than those in the dust samples (Fig. 1f–h).

### Bacterial community structure and bacterial diversity in the filter and dust samples

**Figure 2:**
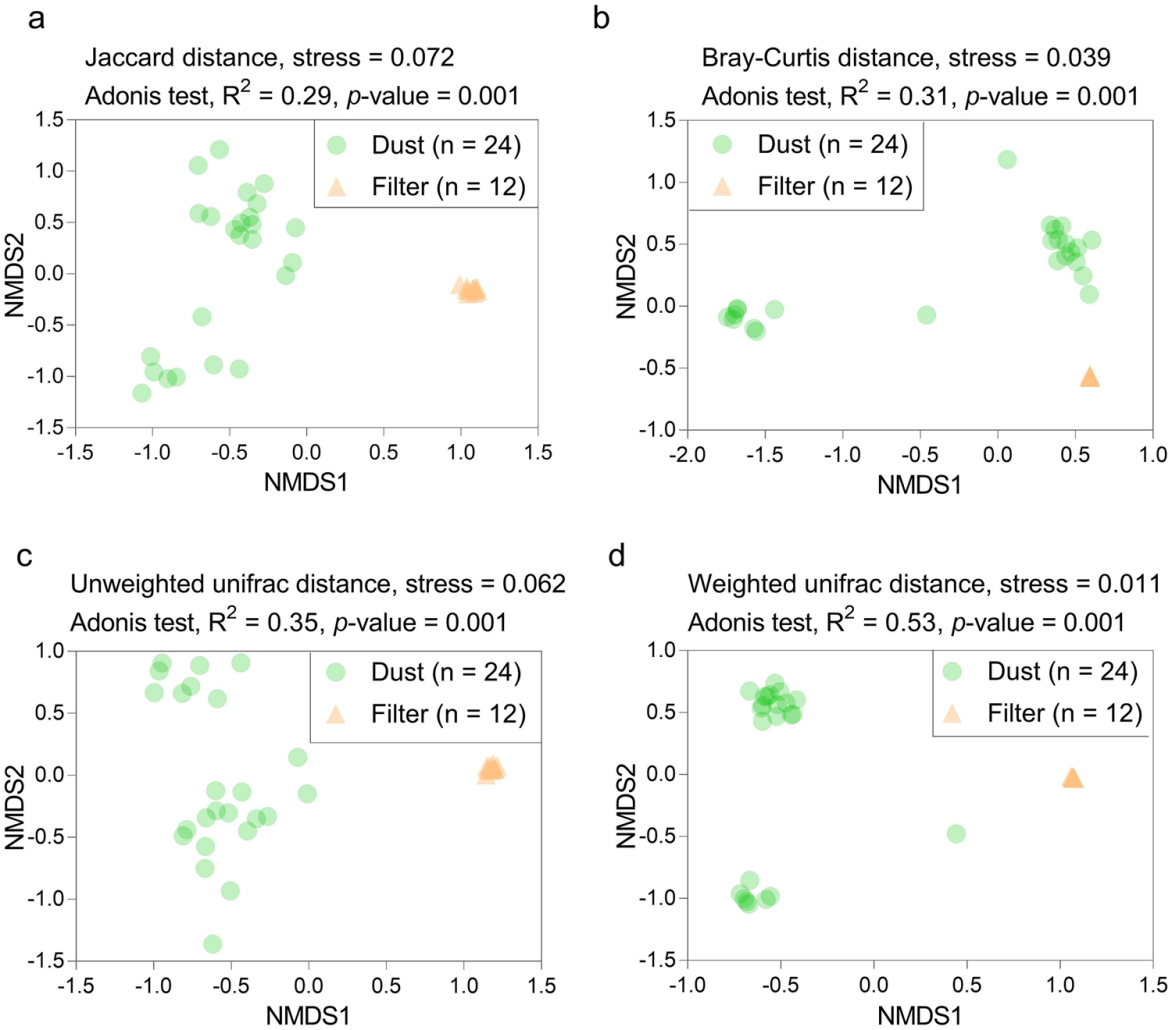
Bacterial community in the dust and filter samples. NMDS analysis based on Jaccard distance (**a**), Bray–Curtis distance (**b**), Unweighted unifrac distance (**c**) and Weighted unifrac distance (**d**). Adonis analysis between the groups was carried out. Stress and the *p*-values are shown in each figure.

**Figure 3:**
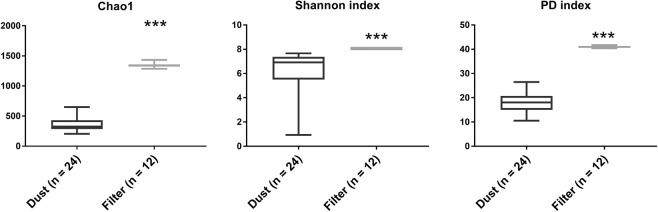
Bacterial diversity in the dust and filter samples—Chao1, Shannon and phylogenetic diversity (PD) index. *Mann–Whitney U test* was carried out. ‘*’: *p* < 0.05, ‘**’: *p* < 0.01, ‘***’: *p* < 0.001.

A total of 836,091 and 526,674 raw reads were obtained separately from 24 dust and 12 filter samples, respectively. Chao1 and Good’s Coverage both showed the sequencing data generally covered most of the species in the samples when they were rarefied to 20,754 sequences (Supplementary Fig. S1). NMDS analysis revealed that the bacterial community structures showed high similarity within filter samples or dust samples (Fig. 2). However, Jaccard, Bray–Curtis, Unweighted unifrac and Weighted unifrac distance analysis revealed significant differences between the filter and dust samples (Fig. 2). There were no significant differences between different rooms (adonis analysis: R = 0.69, *p* = 0.50). A similar bacterial community between rooms was possibly due to a large seasonal fluctuation in the levels of certain microbes, resulting in poor reproducibility^47,48^. In addition, the values of Chao1, Shannon index and phylogenetic diversity (PD) index in the filter samples were significantly greater than those in the dust samples (*p* < 0.05) (Fig. 3).

### Bacterial taxa in the filter and dust samples

**Figure 4:**
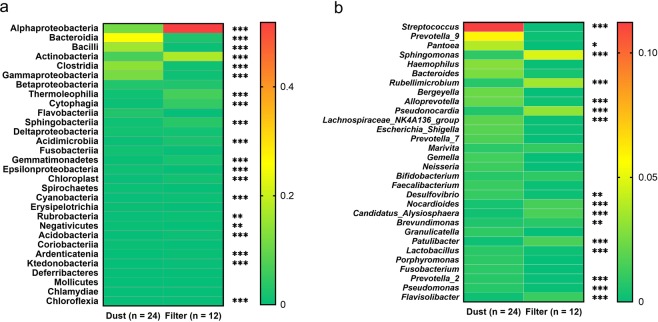
Abundance of top 30 taxa at the class (**a**) and genus (**b**) levels. *Mann–Whitney U test* was carried out in each taxon between dust and filter. The colour represent the mean abundance of each taxon. ‘*’: *p* < 0.05, ‘**’: *p* < 0.01, ‘***’: *p* < 0.001.

The abundance of taxa at the class and genus levels was compared between the filter and dust samples. The abundance of most classes in the filter samples was significantly different from that in the dust samples (Fig. 4a). The major classes in the filter samples were *Alphaproteobacteria* (51.8%) and *Actinobacteria* (17.2%), whereas those in the dust samples were *Bacteroidia* (25.6%), *Clostridia* (13.9%), *Bacilli* (15.9%), *Gammaproteobacteria* (11.7%) and *Alphaproteobacteria* (11.3%) (Fig. 4a). Furthermore, the filter and dust samples showed significant differences in taxa at the genus level. The major genera in the filter samples were *Sphingomonas* (4.7%), *Rubellimicrobium* (3.5%) and *Pseudonocardia* (3.1%), whereas those in the dust samples were *Streptococcus* (11.2%) and *Pantoea* (3.9%) (Fig. 4b).

**Figure 5:**
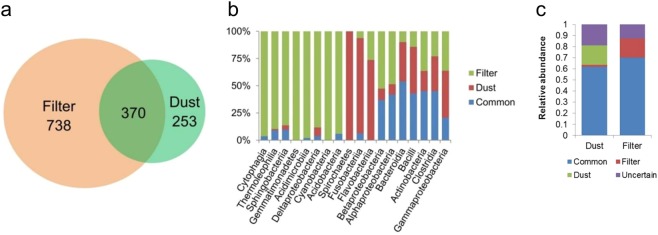
Distribution of operational taxonomic units (OTUs) in the dust and filter. The number of unique and common OTUs in the dust and filter (**a**); the relative proportion of unique and common OTUs in each class (**b**); the abundance of unique and common OTUs in the dust and filter (**c**).

The results of the operational taxonomic units (OTU) distribution showed that the number of unique OTUs in the filter samples was more than twice as much as that in the dust samples (Fig. 5a). Furthermore, most OTU species of *Cytophagia, Thermoleophilia, Sphingobacteriia, Gemmatimonadetes, Acidimicrobiia, Deltaproteobacteria, Cyanobacteria and Acidobacteria* were observed in the filter samples. In contrast, most OTU species of *Spirochaetes, Fusobacteria* and *Flavobacteriia* were observed in the dust samples (Fig. 5b). The common OTUs made up approximately 60% of total reads, and unique OTUs made up 17.1% and 17.6% of total reads in the filter and dust samples, respectively (Fig. 5c).

### Effect of occupancy density on the bacterial community in the filter and dust samples

**Figure 6:**
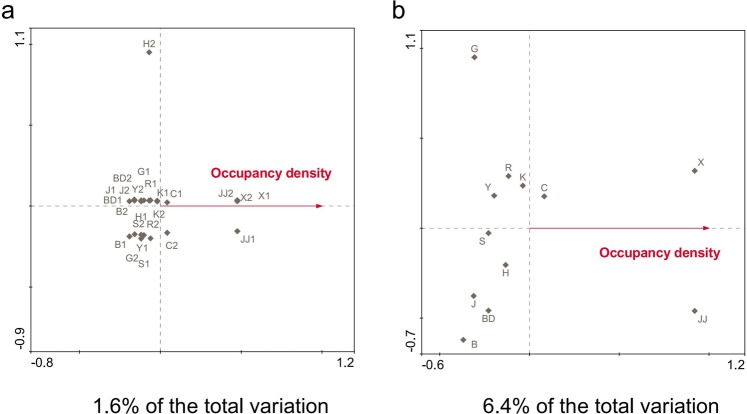
Redundancy analysis of genus assemblage data and occupancy density (red lines) in the dust (**a**) and filter (**b**).

Redundancy analysis (RDA) analysis revealed that 1.6% of the bacterial community in the dust samples and 6.4% of the bacterial community in the filter samples attributed to occupancy density of humans in that room (Fig. 6). Although these values are low, the occupancy density had a greater impact on the bacterial community in the filter samples than that in the dust samples. Furthermore, we found that the abundances of *Bacteroidia* and *Ardenticatenia* in the filter samples significantly positively correlated with occupancy density (*p* < 0.05), whereas *Coriobacteriia* in the dust samples was found to be significantly positively correlated the occupancy density (Table 1). Analysis of the relationship between bacterial composition and occupancy density at the genus level revealed that the abundance of *Klebsiella and Alloprevotella* in the dust and filter samples both significantly positively correlated with occupancy density (Table 1). The taxa at the class and genus levels that were significantly negatively correlated with occupancy density were different in the dust and filter samples (Table 1).

**Table 1:** Spearman correlation analysis between occupancy density and the taxa at class level and genus level in the dust and filter.

		Dust			Filter		
		rho	*p*	Mean	rho	*p*	Mean
				abundance			abundance
Class	*Bacteroidia*	—	—	—	0.79	0.002**	0.024
	*Ardenticatenia*	—	—	—	0.61	0.036*	0.002
	*Coriobacteriia*	0.62	0.032*	0.003	—	—	—
	*Actinobacteria*	−0.64	0.025*	0.064	—	—	—
Genus	*Alloprevotella*	0.62	0.03*	0.022	0.64	0.025*	0.0019
	*Klebsiella*	0.66	0.02*	0.006	0.64	0.026*	0.0001
	*Lentzea*	−0.67	0.018*	0.001	—	—	—

The taxa with mean abundance greater than 0.001 in either group were selected for correlation analysis. Spearman correlation analysis was carried out in stats packages in R environment.

### Bacterial source

**Figure 7:**
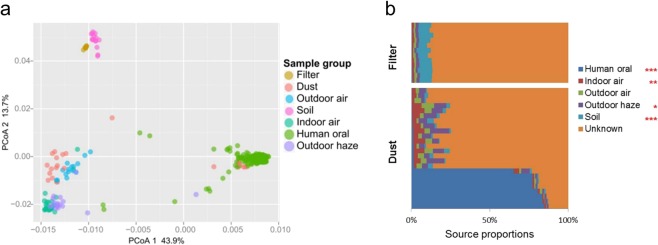
Bacterial source analysis in the dust and filter. Principal coordinates analysis (PCoA) of samples from different sources based on Bray–Curtis distance of bacterial genera (**a**); source tracking using Knight’s method (**b**).

The in-house bacterial community data of outdoor air, soil, indoor air, human oral and outdoor haze (relative works was performed but not published) were used for the principal coordinates analysis (PCoA) analysis. We found that the Bray–Curtis distance between the filter and soil was less than that between the filter and other groups. The bacterial communities of the dust were clustered into two groups: one was similar to outdoor air samples and the other one was similar to human oral bacteria (Fig. 7a). Source tracking was conducted in the R environment based on the Knight’s method^49^. It was found that soil bacteria contributed to a significantly higher proportion of the bacteria in the filter samples than to that in the dust samples (*p* < 0.001); on the other hand, bacteria from human oral, indoor air and outdoor haze contributed to a significantly higher proportion of the bacteria in the dust samples than to that in the filter samples (*p* < 0.001, *p* < 0.01 and *p* < 0.05, respectively) (Fig. 7b). The higher proportion of human oral source as well as indoor air in the separated samples in the dust also suggested that there were two main sources of dust bacteria (Fig. 7b). This is consistent with the findings shown in Fig. 7a.

### Discussion

Bacteria in the environment exist primarily by attaching to living and inanimate surfaces^50^. Two typical inanimate surfaces were analyzed in this research. After attachment to the surfaces, sufficient moisture and nutrients are needed for microbial growth^51^. Floor dust collects the airborne microorganisms around the sampling site by natural settlement, and thus, it can accurately reflect the microbial composition and the microbial levels in indoor air^52^. Indoor bacteria that originate from various sources coexist with PM^40,53,54^. Airborne microorganisms, along with PM, moisture and organic matter from the whole room, are pumped into the air purifier compulsively and intercepted on the filter in an air purifier via its high dust-holding capacity^55,56^. Indoor bacteria are trapped in the fine porous structures of the HEPA filters^55^, but the trapped bacteria are not killed^57^. Atmospheric dust deposited in air filters may serve as a nutrient for microbes^20^. Accompanied by the absorption of organic matter to the filter, the filter changes gradually, forming a more favorable habitat for the microorganisms^57^. The more nutritious habitat might explain the higher proportion of viable bacteria in the filter (Fig. 1).

Two distinct bacterial community types were formed consistent with the sample types (HEPA filter and dust) (Fig. 2). Indoor microbes might be influenced by the outdoor environment owing to the continual flow of outdoor microbes into homes^43,58^ or because indoor environments are incompletely buffered from outdoor microbes^59^. During the usage of the air purifier, indoor air is purified, and particles are enriched on the HEPA film. Meanwhile, a large number of outdoor particles are continuously entering the room. This process enables a large number of outdoor air microorganisms to accumulate on the HEPA film. The types of bacteria in outdoor air can vary according to the function of surrounding soil conditions^60^. Thus, more soil microorganisms are enriched on the HEPA membrane than on the dust because of the high volume of filtered air. The enriched particulate matter membrane helps these microorganisms to survive or reproduce, forming microbial communities similar to the soil environment (Fig. 7a). The bacterial community in floor dust similar to indoor air, outdoor air, outdoor haze and human oral were consistent with main source types^36,43,58,61,62^. Human oral emissions are the largest sources of some floor dust via coughing, sneezing, talking and breathing^63,64^. Among the top 30 genera, more than 10 genera are human-associated bacteria, whereas 5 genera are environmental-associated bacteria. Sources of dominant genera also indicate that humans and the outdoor environment are important bacterial sources in indoor environments^1,65^. The abundance of several human-associated genera was significantly higher in dust samples, including *Streptococcus* (salivary microbiome^66–69^)*, Lachnospiraceae* spp. (human and mammal gut microbiota^70^), *Prevotella* spp. and *Lactobacillus* spp. (vaginal microbiota^71,72^) (Fig. 4). *Sphingomonas* spp. and *Brevundimonas* spp. mainly habitat in HEPA filter, which were ubiquitous in the land habitats^73^ and contaminated environments^74^ (Fig. 4). These were consistent with Fig. 7b. Occupancy density is an important factor affecting the dynamics of the airborne microbial community in the built environment^46^. Its effect on HEPA filter was greater than dust, possibly because of HEPA filter trapped bacteria from indoor air for a much larger volume with a larger amount of human bacteria.

Human oral, indoor air and outdoor haze were critical sources of bacteria in floor dust. In contrast, bacteria present in soil contributed to a significantly higher population in the filter (Fig. 7). The filter in the air purifier and other air-handling equipment could be identified as sources of air contaminants^56,75^. The HEPA filter in the air purifier should be considered as a new ecological niche owing to its enrichment of particles and quite different bacterial sources and bacterial community structure. Air purifiers remove PM by pumping air, and the environmental bacteria are trapped by the HEPA filters^55,57^. Thus, various bacteria possibly tend to keep alive on the filter. Human activities such as walking could greatly increase the emission rate of biological aerosol particles that originated from the surrounding environment^76^. These aerosols may cause adverse effects, including respiratory infections and hypersensitivities in humans^77–79^. Thus, the alive bacteria in the HEPA filter should be considered during the assessment of indoor environmental health.

### Conclusions

Air purifiers are widely used in indoor environments in haze days. Our study showed that the HEPA filters in air purifiers used for a long time were with a higher proportion of viable bacteria compared with the dust. Considering the significantly different bacterial communities and bacterial diversity between the filter and dust samples, HEPA filter in the air purifier should be recognized as a new ecological niche in indoor environment. Source tracking analysis explained the formation of the environment in the filter and dust. The important sources of bacteria were soil and human oral, indoor air and outdoor haze in the case of filter and dust, respectively. The number of unique OTUs in the filter samples was more than twice as much as that in the dust samples. The unique OTUs made up 17.1% and 17.6% of the total reads in the filter and dust samples, respectively. Our study suggested that air purifiers should be included in the evaluation of indoor environmental health risks.

## Methods

### Site description and sample collection

Twelve office rooms were selected as the sampling rooms in the Institute of Laboratory Animal Science (ILAS) in Beijing, China. These rooms were used as administrative offices that were not related to laboratory and animal houses. These rooms were distributed in three different buildings, and each room was independent of each other. Each room had openable windows. Ventilation was carried out by opening windows in no haze day. All the rooms were covered with tiles. Different types of house dust samples are widely used to assess the health effects of indoor microbes representing airborne inhalation exposure. Although it was reported a better reproducibility of microbial determinations has been found for mattress dusts^80,81^, settled dust was suitable to study the microbes in the office room. Settled dust reflects the microbial composition of indoor air and responds similarly to environmental determinants^36^. In March 2016, two randomly different areas on these room floors, which were larger than 2 × 2 cm^2^ with little or no direct contact with the occupants, were disinfected with bromogeramine and marked as floor dust sampling sites, and a new HEPA filter was set in the air purifier in each of these rooms. The flow rate of the air purifier was 116 m^3^/h. The air purifiers were applied in haze day in which a 24-hour average concentration was greater than 75 μg/m^3^. The sampling rooms and the occupant numbers in each room are shown in Supplementary Table S1. Twenty-four dust sampling sites were left undisturbed, and 12 air purifiers were used without replacing the filter for 1 year. The air purifier used days in each room were different, owing to differed office plans and arrangements. The range was 121–143 days, except for holidays. In March 2017, dust samples were collected with disposable sterile gloves and masks from the 24 dust sampling sites. A sterile cotton swab was used with 100 µL sterile physiological saline (salt content: 0.9%) to smear the floor dust and kept in 1.5 mL sterile Eppendorf tubes at −80 °C before using them for DNA isolation. The filter samples were collected with disposable sterile gloves and masks from the 12 air purifiers (Supplementary Fig. S2). The concentrations of PM_2.5_ and PM_10_ in Beijing from March 2016 to March 2017 are shown in Supplementary Fig. S3. Maestre *et al*. found that vacuum and swab samples of HVAC filters were repeatable and generally superior to cut samples^28^. We thought it was easy to handle the filter and control operation consistency by cut. Furthermore, the dust recovery methodologies did not affect the contribution of environmental and human sources to the bacterial and fungal communities in HVAC filter^28^. Thus, we selected to sample by cut. A 2 × 2 cm^2^ area of the outer membrane filter was sheared and cut with sterile surgical scissors. The filter fragments were placed in 5 mL of saline and vortexed for 20 min. The supernatants were pipetted out and centrifuged at 13,000 rpm for 2 min. Subsequently, the supernatants were removed, and the pellets were maintained in 1.5 mL sterile Eppendorf tubes at −80 °C before DNA isolation.

Samples from outdoor air, soil, indoor air, human oral and outdoor haze were used to study the source of bacteria in the filter and dust. The detailed information of the samples is presented in Table 2. Sample sequencing steps were similar to those performed for the filter and dust samples.

**Table 2:** The sample information used in source tracking analysis.

Group	Source	Sampling method	Sampling process	Sampling time	Sampling sites
Indoor air	indoor air	Natural sedimentation	Place uncovered, disposable, sterile cell culture dishes (φ90 mm) at a height of 1.5 m above the ground and left them for 12 h, Samples were then collected by smearing the inner surface of each dish with a sterile swab that had been dipped in 100 μL of sterile normal saline, and the swabs were used for DNA extraction.	December 9 to 27, 2016	Bei Jing
Outdoor haze	outdoor haze	Natural sedimentation	At 8:00 and 20:00, 200 ml deionized water was used for mouthwashes collection. The samples were centrifuged with 12000 g for 10 min, and the deposit was used for DNA extraction.		
Human oral	human oral	Mouthwashes			
Outdoor air	outdoor air	Air impact method	3000 L air was collected with Planktonic airborne bacteria sampler FKC1 with flux of 100 L/min onto the surface of sterile cell culture dishes covered with 200 ul 20% glycerol. Samples were then collected by smearing the inner surface of each dish with a sterile swab that had been dipped in 100 μL of sterile normal saline, and the swabs were used for DNA extraction.	September 12 to 13, 2017	Shi Jiazhuang
Soil	soil	Digger blade	Remove 5 cm of topsoil with disposable sterile shovel, and collect 20 g soil with sterile 50 ml tube. The soil was used for DNA extraction		

### Cell viability analysis of the dust and filter samples

The cell viability of the samples was determined by Invitrogen™ LIVE/DEAD^®^ BacLight^TM^ Bacterial Viability kits, which was used to evaluate the cell membrane integrity^82^. These kits utilize mixtures of the SYTO^®^ 9 green-fluorescent nucleic acid stain and the red-fluorescent nucleic acid stain, propidium iodide. Bacterial suspensions from dust and filter samples were obtained by mixing the bacterial pellet with 100 µL saline. The *Escherichia coli* (*E*. *coli*) suspension with 100%, 50% and 0% live cells was prepared as positive controls, and samples without dyeing were prepared as a negative control. The remaining steps were performed as directed by the manufacturer.

### DNA extraction and Illumina sequencing of 16S rRNA genes

Genomic DNA was extracted from the dust and filter samples by using the PowerSoil^®^ DNA Isolation Kit (MO BIO Laboratories, Inc., Carlsbad, CA, USA) according to the manufacturer’s recommendations. The quality and quantity of DNA were verified by NanoDrop and agarose gel electrophoresis (Table S1). There was no significant difference in DNA content between HEPA filter and floor dust (Fig. S4). The extracted DNA was diluted to a concentration of 1 ng/μL and stored at −20 °C until further processing. The diluted DNA was used as the template for PCR amplification (26 cycles) of bacterial 16 S rRNA genes with barcoded primers and HiFi Hot Start Ready Mix (KAPA). For the bacterial diversity analysis, the V3–V4 variable region of the 16 S rRNA genes was amplified with universal primers (343 F 5′-TACGGRAGGCAGCAG-3′, 798 R 5′-AGGGTATCTAATCCT-3′)^83^. The length of the PCR product was about 445 bp. Negative control was used in the same amplification system by using sterile deionized water as the template to monitor the contamination of the amplification system. The amplicon quality was visualized by gel electrophoresis, followed by the purification of PCR product with AMPure XP beads. Amplification for another round of PCR (7 cycles) was conducted, and the product was purified again. In each batch, we ran negative controls. We monitored the PCR systems to avoid contamination of the PCR product with undesirable DNA. If no 400 bp band was detected in the negative control, the samples in the same batch were processed to the sequencing steps. The final amplicon concentration was then quantified using the Qubit™ dsDNA Assay Kit. Equal amounts of the purified amplicons were pooled for subsequent sequencing with MiSeq Reagent Kit v2 (Illumina, Inc., San Diego, CA, USA) using the MiSeq Sequencing System (Illumina, Inc., San Diego, CA, USA). The PE300 sequencing model was used, and paired ends were applied.

### Binning sequences into species

For further taxonomy analysis, the community-by-operational taxonomic unit (OTU) matrix was generated as follows. The raw sequencing data after Illumina sequencing were in the FASTQ format. The paired-end reads were then pre-processed using the Trimmomatic software to detect and cut off ambiguous bases (N)^84^. It also cuts off low-quality sequences with average quality scores below 20 by using the sliding window trimming approach. After trimming, the paired-end reads were assembled using the FLASH software^85^. The parameters of the assembly were as follows: 10 bp of minimal overlapping, 200 bp of maximum overlapping, and 20% of the maximum mismatch rate. Further denoising of the sequences was performed as follows: reads with ambiguous sequences, homologous sequences, or sequences below 200 bp were abandoned. Reads with 75% of the bases above Q20 were retained. Then, reads with the chimera were detected and removed^86^. These two steps were achieved using the QIIME software (version 1.8.0)^87^.

Clean reads were subjected to primer sequence removal and clustering to generate OTUs using the UPARSE software with 97% similarity cutoff^88^. The representative read of each OTU was selected using the QIIME package. All the representative reads were annotated and blasted against the Silva database Version 123 (16 S rDNA) using the RDP classifier (confidence threshold was 70%)^89^. Each sample was rarefied to 20,754 sequences.

### Bacterial diversity and other statistical analyses

To ensure sampling depth, the Chao1 and Good’s Coverage Estimator was calculated to analyze the species diversity in each sample. The α-diversity^90^ of the bacterial community was analyzed on the basis of Chao1, Shannon–Wiener and Faith’s phylogenetic diversity (PD)^91^ index using the new OTU matrix file. The phylogenetic tree was built using approximately maximum likelihood based on the evolutionary distance calculated by Jukes and Cantor model with QIIME. The phylogenetic tree was used for PD index, Unweighted unifrac and Weighted unifrac distance analysis. We performed non-metric multi-dimensional scaling (NMDS) analysis^92^ to compare the bacterial community structure in the filter and dust based on Jaccard, Bray–Curtis, Weighted and Unweighted unifrac distances 92. Permutational multivariate analysis of variance (Adonis) was also carried out using the package ‘vegan’ in the R environment. All other statistical analyses were performed using the package ‘state’ in the R environment.

The dominant 30 taxa at the class and genus level were used to examine the differences of bacterial composition between the dust and filter. Mann–Whitney U test was performed, and *p-values* less than 0.05 were considered statistically significantly different. A unique OTU distribution was also analyzed. The OTU that existed in more than three samples in dust and less than three samples in the filter was considered as unique in the dust. The OTU that existed in more than three samples in the filter and less than three samples in dust was considered as unique in the filter.

Human occupants are an important source of indoor microorganisms. We performed a Spearman correlation analysis between DNA content and room area, the number of people and occupancy density and found no correlation (Supplementary Table S2). We explored the Spearman correlation analysis between taxa (comprising > 0.1%) at the class and genus level and occupancy density. To compare the differences in the effect of occupancy density on the bacterial community in filter and dust, RDA was performed for dust and filter samples using CANOCO 4.5 software (http://canoco.software.informer.com/4.5/). In this study, dominant genera (comprising > 0.1%) were used to analyze the correlations with occupancy density by RDA. A PCoA was performed based on the Bray–Curtis distance of genera among samples from different sources. Source tracking was carried out in the R environment based on the Knights’ method^49^.

All bacterial 16 S rRNA gene sequences generated for the present study were deposited in the National Centre for Biotechnology Information Sequence Read Archive (http://www.ncbi.nlm.nih.gov/sra) under the accession number SRP160378.

## Supplementary information

Supplementary information.

## Data Availability

All bacterial 16 S rRNA gene sequences generated for the present study have been deposited in the National Centre for Biotechnology Information Sequence Read Archive (http://www.ncbi.nlm.nih.gov/sra) under the accession number SRP160378.
