# Author Correction: Bacterial community analysis of floor dust and HEPA filters in air purifiers used in office rooms in ILAS, Beijing

**DOI:** 10.1038/s41598-020-67049-8

**Published:** 2020-06-23

**Authors:** Jianguo Guo, Yi Xiong, Taisheng Kang, Zhiguang Xiang, Chuan Qin

**Affiliations:** 1 NHC Key Laboratory of Human Disease Comparative Medicine, Institute of Laboratory Animal Sciences, CAMS&PUMC, Beijing, 100021 China; 2 Key Laboratory of Human Diseases Animal Model, State Administration of Traditional Chinese Medicine, Beijing, 100021 China; 3 Department of Food Science and Engineering, School of Chemistry and Chemical Engineering, Harbin Institute of Technology, Harbin, 150001 China

Correction to: *Scientific Reports* 10.1038/s41598-020-63543-1, published online 14 April 2020

In the original version of this Article, there were errors in Affiliations 1 and 2 which were incorrectly listed as ‘Institute of Laboratory Animal Science, Chinese Academy of Medical Sciences & Peking Union Medical College, Beijing, 100021, China’ and ‘NHC Key Laboratory of Human Disease Comparative Medicine, Beijing, 100021, China’ respectfully.

The correct affiliations are listed below.

Affiliation 1

NHC Key Laboratory of Human Disease Comparative Medicine, Institute of Laboratory Animal Sciences, CAMS&PUMC, Beijing 100021, China

Affiliation 2

Key Laboratory of Human Diseases Animal Model, State Administration of Traditional Chinese Medicine, Beijing 100021, China.

This error has now been corrected in the PDF and HTML versions of the Article, and in the accompanying Supplementary Information file.

