# Supplementary material for: Bacterial community analysis of floor dust and HEPA filters in air purifiers used in office rooms in ILAS, Beijing
Source: Sci Rep. 2020 Apr 14;10:6417. doi: 10.1038/s41598-020-63543-1 (PMC7156680; doi:10.1038/s41598-020-63543-1)
Supplement: Supplementary file 1 — Supplementary information. [file 41598_2020_63543_MOESM1_ESM.docx]

**Supplementary content for:**

**Bacterial community analysis of floor dust and HEPA filters in air purifiers used in office rooms in ILAS, Beijing**

Jianguo Guo^1,2^, Yi Xiong^3^, Taisheng Kang^1,2^, Zhiguang Xiang^1,2^, Chuan Qin^1,2*^

^1^Institute of Laboratory Animal Science, Chinese Academy of Medical Sciences & Peking Union Medical College, Beijing 100021, China.

^2^NHC Key Laboratory of Human Disease Comparative Medicine, Beijing 100021, China.

^3^Department of Food Science and Engineering, School of Chemistry and Chemical Engineering, Harbin Institute of Technology, Harbin 150001, China

| a |
| --- |
| 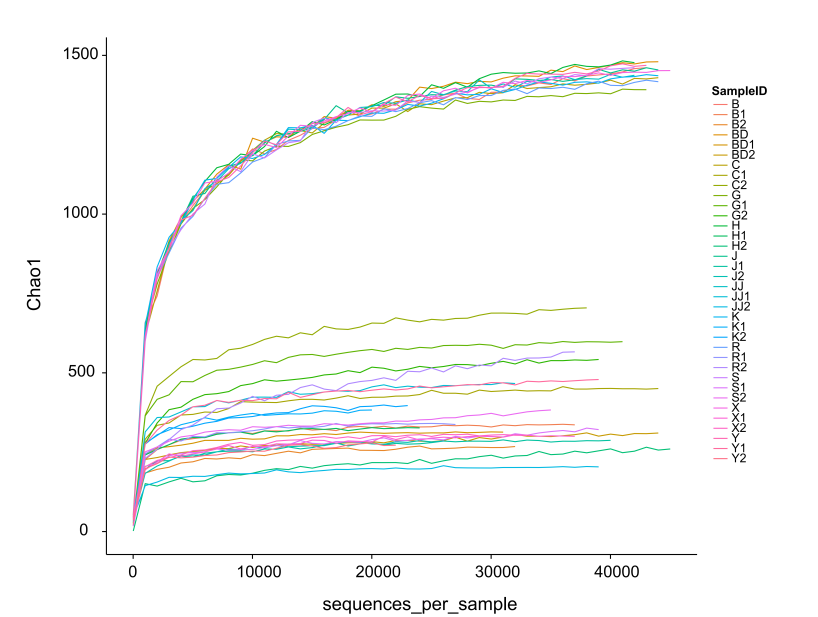 |
| b |
| 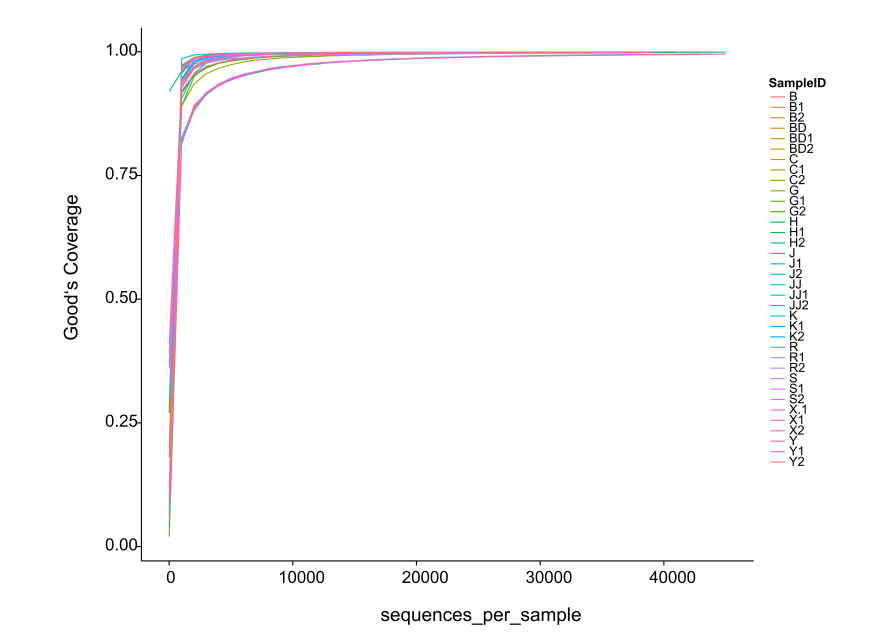 |

**Figure S1.** Values of Chao1 (a) and Good’s Coverage (b) of sequencing samples with different sequences


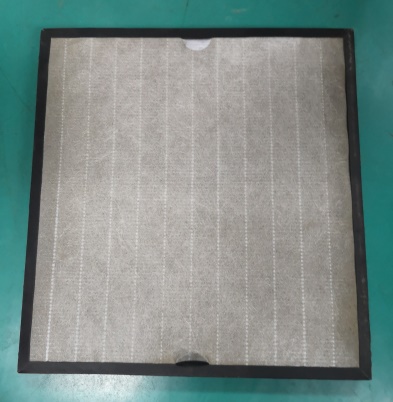
**
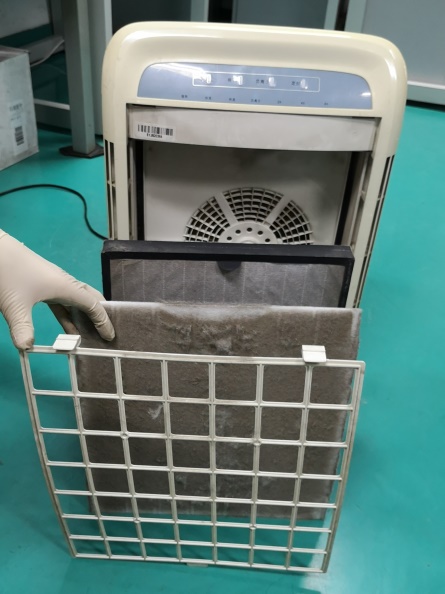
**

**Figure S2.** Air purifier used in our study and the outer layer of the HEPA filter we focused on

**Figure S3.** The concentration of PM_2.5_ and PM_10_ in Beijing from March 2016 to March 2017

**Figure S4.** DNA content in HEPA filter and floor dust

**Table S1.** Information of samples

| Sample | Sample type | Group | Sampling room | Occupants  number | Area (m^2^) | Occupancy density (people /m^2^) | DNA content  (ng/cm^2^) |
| --- | --- | --- | --- | --- | --- | --- | --- |
| BD1 | dust | Dust | Room BD | 5 |  |  | 21 |
| BD2 | dust |  |  |  | 12 | 0.42 | 14 |
| BD | filter | Filter |  |  |  |  | 70 |
| B1 | dust | Dust | Room B | 13 |  |  | 135 |
| B2 | dust |  |  |  | 16 | 0.81 | 143 |
| B | filter | Filter |  |  |  |  | 45 |
| C1 | dust | Dust | Room C | 2 |  |  | 88 |
| C2 | dust |  |  |  | 10 | 0.20 | 151 |
| C | filter | Filter |  |  |  |  | 35 |
| G1 | dust | Dust | Room G | 8 |  |  | 28 |
| G2 | dust |  |  |  | 14 | 0.57 | 23 |
| G | filter | Filter |  |  |  |  | 53 |
| H1 | dust | Dust | Room H | 5 |  |  | 130 |
| H2 | dust |  |  |  | 16 | 0.31 | 174 |
| H | filter | Filter |  |  |  |  | 40 |
| JJ1 | dust | Dust | Room JJ | 1 |  |  | 10 |
| JJ2 | dust |  |  |  | 12 | 0.08 | 11 |
| JJ | filter | Filter |  |  |  |  | 28 |
| J1 | dust | Dust | Room J | 7 |  |  | 178 |
| J2 | dust |  |  |  | 12 | 0.58 | 35 |
| J | filter | Filter |  |  |  |  | 40 |
| K1 | dust | Dust | Room K | 5 |  |  | 5 |
| K2 | dust |  |  |  | 20 | 0.25 | 23 |
| K | filter | Filter |  |  |  |  | 53 |
| R1 | dust | Dust | Room R | 3 |  |  | 18 |
| R2 | dust |  |  |  | 10 | 0.30 | 44 |
| R | filter | Filter |  |  |  |  | 40 |
| S1 | dust | Dust | Room S | 5 |  |  | 28 |
| S2 | dust |  |  |  | 12 | 0.42 | 29 |
| S | filter | Filter |  |  |  |  | 23 |
| X1 | dust | Dust | Room X | 1 |  |  | 279 |
| X2 | dust |  |  |  | 12 | 0.08 | 21 |
| X | filter | Filter |  |  |  |  | 60 |
| Y1 | dust | Dust | Room Y | 6 |  |  | 56 |
| Y2 | dust |  |  |  | 16 | 0.38 | 18 |
| Y | filter | Filter |  |  |  |  | 53 |

**Table S2.** Spearman correlation analysis between DNA content and room parameters

|  |  | Room area | Number of people | Occupancy density |
| --- | --- | --- | --- | --- |
| Dust | rho | -0.03 | 0.11 | 0.09 |
| Filter | rho | 0.27 | 0.14 | 0.09 |

Spearman correlation analysis was carried out with corr.test in the package of psych in R environment
